# Fuzzy-Inspired Photoplethysmography Signal Classification with Bio-Inspired Optimization for Analyzing Cardiovascular Disorders

**DOI:** 10.3390/diagnostics10100763

**Published:** 2020-09-28

**Authors:** Sunil Kumar Prabhakar, Harikumar Rajaguru, Sun-Hee Kim

**Affiliations:** 1 Department of Brain and Cognitive Engineering, Korea University, Anam-dong, Seongbuk-gu, Seoul 02841, Korea; sunilprabhakar22@gmail.com; 2 Department of Electronics and Communication Engineering, Bannari Amman Institute of Technology, Sathyamangalam 638402, India; harikumarrajaguru@gmail.com

**Keywords:** PPG, feature extraction, cardiovascular levels, optimization, classification

## Abstract

The main aim of this paper is to optimize the output of diagnosis of Cardiovascular Disorders (CVD) in Photoplethysmography (PPG) signals by utilizing a fuzzy-based approach with classification. The extracted parameters such as Energy, Variance, Approximate Entropy (ApEn), Mean, Standard Deviation (STD), Skewness, Kurtosis, and Peak Maximum are obtained initially from the PPG signals, and based on these extracted parameters, the fuzzy techniques are incorporated to model the Cardiovascular Disorder(CVD) risk levels from PPG signals. Optimization algorithms such as Differential Search (DS), Shuffled Frog Leaping Algorithm (SFLA), Wolf Search (WS), and Animal Migration Optimization (AMO) are implemented to the fuzzy modeled levels to optimize them further so that the PPG cardiovascular classification can be characterized well. This kind of approach is totally new in PPG signal classification, and the results show that when fuzzy-inspired modeling is implemented with WS optimization and classified with the Radial Basis Function (RBF) classifier, a classification accuracy of 94.79% is obtained for normal cases. When fuzzy-inspired modeling is implemented with AMO and classified with the Support Vector Machine–Radial Basis Function (SVM–RBF) classifier, a classification accuracy of 95.05% is obtained for CVD cases.

## 1. Introduction

By utilizing the infrared light at the peripheral parts of the body, cardiovascular function can be assessed by a famous non-invasive technique known as Photoplethysmography (PPG) [1]. In PPG sensors, the source is generally a Light-Emitting Diode (LED), and the detector is generally a Light-Dependent Resistor (LDR) that operates in the infrared range (0.8–1 μm) [2]. The fundamental difference in the infrared light absorbance by the blood and the rest of the skin tissues determines the sensing of the PPG [3]. Cardiovascular parameters such as cardiac output, blood pressure, heart rate, blood oxygen saturation, respiratory rate, and vascular function can be analyzed with the help of PPG waveform [4]. CVD is one of the serious health issues among the world population as it contributes to a high mortality rate [5]. Various risk factors contributing to it are smoking, obesity, physical inactivity, stress, alcoholism, high cholesterol levels, etc. [6]. To mitigate the total number of deaths due to CVD cases, continuous evaluation by medical professionals should be done on a regular basis [7]. For monitoring the physiological conditions of a particular patient, one of the most famous technologies utilized is PPG, as it is non-invasive in nature and is inexpensive, too. As a result of its capacity to perform continuous readings, PPG can usually be applied to pulse oximetry readings [8]. The essential information about both cardiovascular and respiratory systems is provided by this signal. The PPG has a vast viability, and it is easy to utilize the signal for research activities. The PPG signal also does not have a complex implementation in terms of hardware when it is compared to the Electrocardiogram (ECG) signal [9]. Even a reference signal is not required for PPG, thereby making the PPG sensors able to be incorporated with wristbands. Thus, the application, utility, or results and their clinical applicability is too good and can be used in various research for the analysis and diagnosis of CVD [10].

Previous works in the aspects of analysis of PPG [11], classification of PPG [12], artifact reduction of PPG [13], cardiac arrhythmia classification of PPG [14], heart rate monitoring from PPG [15], and development of PPG sensors [16] have been reported in the literature. The automatic Region of Interest (ROI) for remote PPG was performed by Gallego and Haan [17]. Utilizing repeated Gaussian filters and cross-correlation, the PPG signal quality estimation was done by Karlen et al. [18]. For PPG, an algorithm for real-time pulse waveform segmentation with artifact detection was done by Fischer et al. [19]. Multiple regression analysis along with neural networks was utilized to estimate the blood pressure variation with PPG signals by Cho et al. [20]. The PPG signal motion artifact modeling for the purpose of heart rate monitoring using wearable devices was done by Cajas et al. [21]. The exposure of heart rate variability through PPG analysis was analyzed quantitively in motor bike riders by Ramasamy et al. [22]. The automated discrimination between hypovolemia and euvolemia was done on PPG signals using Support Vector Machine (SVM) by Reljin et al. [23]. For interpreting Cardiovascular Disorders (CVD), a metaheuristic-based dimensionality reduction and classification analysis of PPG signals was performed by Prabhakar et al. [24]. During maximal exercise test, a heuristic algorithm for tracking photoplethysmographic heart rate was done by Silva et al. [25].

As far as fuzzy logic is concerned, its application has been enormous. Fuzzy algorithms are not sensitive to the changing surroundings and are less erroneous, thereby making them robust. When comparing the computationally precise system, the reasoning process is quite simple, and so a lot of computing power can be saved in incorporating fuzzy methods. A fuzzy inference system was developed to identify Event-Related Desynchronization (ERD) for Brain–Computer Interface (BCI) applications [26]. A fuzzy logic traffic signal controller was optimized through the differential evolution algorithm for various traffic scenarios by Dogan and Akgungor [27]. A fuzzy rule-based system was utilized for detecting and visualizing stress during commuter driving by Dobbins and Fairclough [28]. A fuzzy optimization concept was utilized by Prabhakar and Rajaguru along with Modified Adaboost Classifier for epilepsy classification [29]. Fuzzy techniques were utilized by Prabhakar and Rajaguru to analyze the cerebral blood flow for epileptic and diabetic patients [30]. As far as the fuzzy-inspired photoplethysmography signal classification is concerned, Liu et al. [31] proposed a work where a fuzzy logic discriminator was utilized for heart rate extraction from PPG signals. The fuzzy logic was utilized to authenticate PPG signals by Gu and Zhang [32]. For the ubiquitous healthcare, the detection of heartbeat based on filter banks and fuzzy inference was done by Lee and Kang [33]. For improving the stroke volume measurement, the PPG signal quality was classified using fuzzy neural networks by Liu et al. [34]. Not much literature is available with exception to the very few works done with the application of fuzzy concept to PPG signal modeling, analysis, or classification. Other than that, the methodology proposed in this paper is first of its kind utilized for the efficient classification of CVD. The organization of the paper is as follows. In Section 2, the materials and methods are explained, followed by the fuzzy-inspired Modeling in Section 3. Section 4 explains the different types of optimization utilized here for selecting the best values and optimizing it followed by the classification in Section 5. It is followed by the results and discussion in Section 6 and conclusion in Section 7.

## 2. Materials and Methods

**Figure 1:**
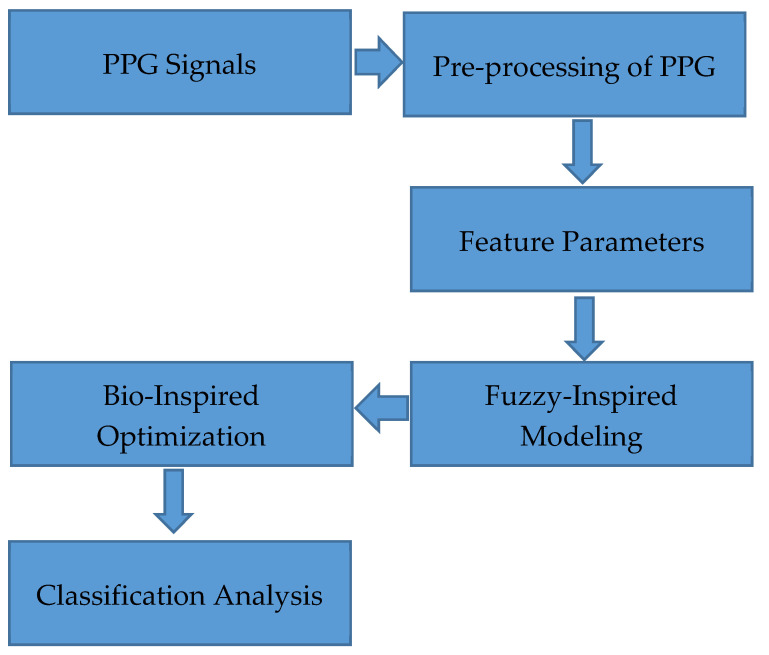
Block diagram of the work.

From the Capnobase dataset, the various morphological waveforms have been obtained from the IEEE TMBE Pulse Oximetry database [35]. The raw PPG signal recordings of 8-minute duration have been found in this dataset. In this database, the annotated representation of signals such as Capnogram (inclusive and exclusive CO_2_, pressure, and respiratory flow) are present. In the Capnobase dataset, the entire IEEE benchmark (42 records) has been considered for the experiment with 28 records representing the CVD and 14 records representing the normal condition. A 100 Hz sampling rate was utilized in this PPG dataset. About 150,000 samples per patient are obtained with this data length, and it is preprocessed with the help of Independent Component Analysis (ICA). The block diagram of the work is shown in Figure 1.

## 3. Fuzzy-Inspired Modeling

To manipulate noisy and imprecise information and to obtain decisions based on such data, one of the most effective tools is fuzzy set theory [36]. A linguistic approach can be offered by fuzzy systems so that a quite reasonable inference can be made. The fuzzy modeling for PPG risk levels at every two-second segment from PPG signals are analyzed. The optimization of the segment results is performed, as they are at various risk levels. Once the optimization is achieved, the fuzzy modeling is performed. The different parameters obtained after sampling are provided as inputs to the fuzzy system. The parameters obtained from PPG signals here are Energy, Variance, Approximate Entropy (ApEn), Mean, Standard Deviation, Skewness, Kurtosis, and Peak Maximum.

### 3.1. Fuzzy Membership Functions

Initially, energy is considered to be a prominent parameter, and the other seven input features are analyzed with it to get seven outputs. Five linguistic levels such as Normal (N), Low (L), Medium (M), High (H), and Very High (VH) are utilized with each input feature. For representing the linguistic levels of the Energy, Variance, ApEn, Mean, Standard Deviation, Skewness, Kurtosis, and Peak Maximum, triangular membership functions are defined. The classification of the output risk level is also classified into five linguistic levels such as Normal (N), Low (L), Medium (M), High (H), and Very High (VH). The representation of risk level classifications of a PPG signal is given in Table 1. The binary representation of risk levels is given in Table 2. For each representation, a binary string is associated, and its respective weight and probability is calculated. The parameter ranges for various risk levels are given in Table 3. The Fuzzy Associative Matrix (FAM) table is expressed in Table 4.

Table 1 shows the fuzzy linguistic sets to represent the output risk levels of the subject through PPG signal parameters. The five level of cardiac risk levels will vary from Normal as represented by the string A to Very High risk level as represented by string E.

Table 2 shows the binary representation of cardiac risk levels to represent the fuzzy modeled outputs in the coded form along with their positional values. The output fuzzy values are processed as an individual code by a specific coding method. Since working on definite alphabets is much easier and hassle-free than processing numbers with large decimal accuracy, the output is encoded with a specific string of alphabets. The alphabetical representation of the five classifications of the outputs is shown in Table 1. These numerical values are associated with the probability of each coded CVD risk-level pattern. The five risk levels are encoded in descending order as E > D > C > B > A in binary strings of five-bit length using a weighted positional indication, as shown in Table 2. A string of seven letters named chromosomes is obtained by encoding each output risk level of the fuzzy output, the value of which is computed as the total sum of probabilities of the individual specific genes. For example, if the output of an epoch is encoded as EEDDCBE, its value would be 0.322577. Now, each fuzzy modeled pattern is encoded in the numerical form of the range 0–1.

Table 3 indicates the edges of the triangular membership functions associated with the five linguistic fuzzy sets in the type I fuzzy system for the eight derived parameters from the PPG signal samples. As shown in the Table 3, the linguistic fuzzy sets are overlapped with the adjacent fuzzy sets. This is clearly depicted in the FAM shown in Table 4.

As indicated in the Table 4, the FAM table for energy vs. variance parameters shows the overlapping of the fuzzy membership functions along with the adjacent linguistic sets only. Therefore, we can effectively utilize the thirteen fuzzy rules instead of twenty-five fuzzy rules. In the Table 4, x indicates the “do not care” condition.

### 3.2. Fuzzy Rule Set

The fuzzy rules in this work are framed in the format as:

- If Energy is Low and Variance is Low, then the Output is of Low-Risk Level
- If Energy is High and Variance is Medium, then the Output is of High-Risk Level

In this fuzzy system (2 × 1), two inputs and one output are present. We have five linguistic levels of energy and five linguistic levels of other seven features such as Variance, ApEn, Mean, Standard Deviation, Skewness, Kurtosis, and Peak Maximum. Therefore, there are seven individual fuzzy systems available. Therefore, we obtain a total rule base of 175 rules based on seven sets, each comprising 25 rules. This is a type of exhaustive fuzzy rule-based system developed to get the perfect results. The fuzzy modeled output for a two-minute duration of PPG signals in the CVD case is expressed in Table 5.

**Figure 2:**
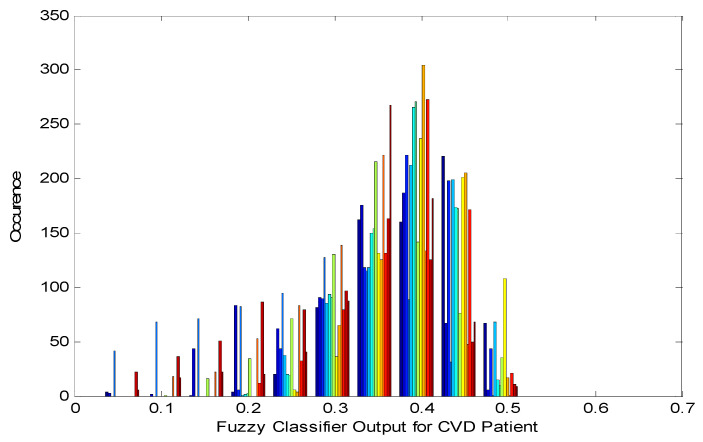
Histogram of fuzzy modeled output.

BEEEEED corresponds to two hundred samples of two-second duration in the PPG signal. There are sixty such code words shown in Table 5 (12 × 2 × 5 = 120 s). The target code in this case is EEEEDEE = 0.47925. Likewise, the fuzzy model will produce 750 code words per patient, and all the words highly differ among themselves. Therefore, a mechanism to identify the diversity of the fuzzy modeled outputs can be further exhibited. The histogram of the fuzzy modeled output is shown in Figure 2.

**Figure 3:**
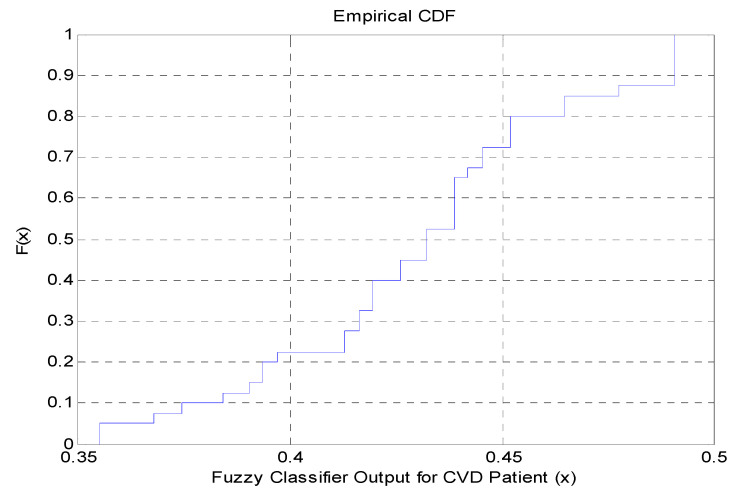
Cumulative Density Function (CDF) plot of the fuzzy modelled output.

Figure 2 shows the presence of discontinuity points in the attained code words of the fuzzy system. This is further authenticated by the Cumulative Density Function (CDF) plot of the fuzzy modeled system, as shown in Figure 3. As demonstrated in Figure 3, the fuzzy modeled outputs are highly discontinued and have a step-level pattern or representation.

**Figure 4:**
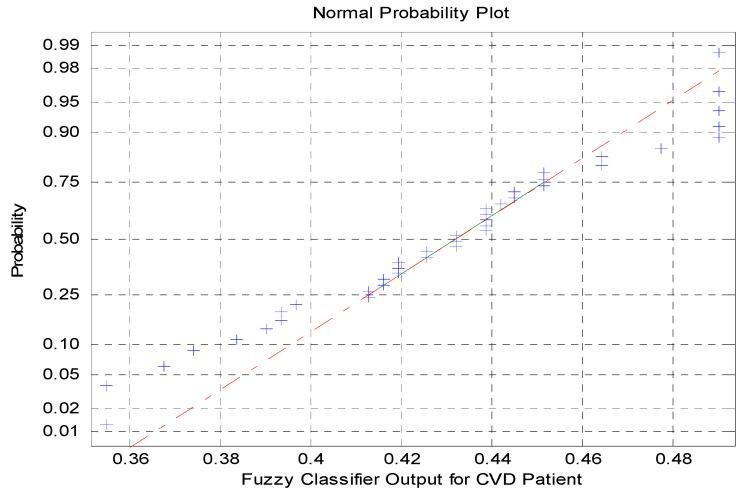
Normal probability plot for the fuzzy modeled output.

The Normal Probability Plot for the fuzzy modeled output is shown in Figure 4, and it indicates the outliers in the code words of the fuzzy modeled output. Therefore, it is necessary to optimize the fuzzy modeled outputs to attain a singleton pattern, which in turn represents the cardiac risk level of the patient.

Furthermore, to identify the presence of non-linearities in the fuzzy modeled outputs, it can be explained by the estimation of Rhythmicity and Hurst exponent parameters. Table 6 shows the analysis of the Rhythmicity and Hurst exponent for fuzzy modeled outputs.

(1) $$R=\frac{C}{D}$$

where *R* = Rhythmicity, *C* = No. of categories of patterns; and *D* = Total number of patterns, which is 21,000 in CVD cases and for Normal cases, *D* = 10,500. For an ideal classifier C to be considered one, *R* = 4.76E-05 for CVD cases and *R* = 9.52381E-05 as in the consideration of normal cases. However, we attained *C* = 2161 for CVD cases and *C* = 1172 for normal cases. Table 6 shows the higher rhythmicity value of fuzzy modeled outputs, which implies that the fuzzy model needs further optimization to produce a singleton output. In a time series, the degree of long-range dependence, predictability, and self-similarity is assessed by the Hurst exponent (H). Dependent on asymptotic behaviour, it also means the smoothness of a fractal time series. For various types of signals, the value of a Hurst exponent is specified as follows. The Hurst exponent value of 0.5 matches a truly random time series. Anti-persistent behavior is exhibited by the Hurst exponent if 0 < H < 0.5. The duration for every sample must be changed in the time series at the limit of H = 0. A temporarily persistent time series is described by the Hurst exponent if 0.5 < H < 1. As shown in the Table 6, the Hurst exponent values are distinct in the two classes of the fuzzy modeled outputs.

## 4. Optimization Techniques

Optimization techniques have been implemented in many areas to handle different practical issues [37]. In different engineering and medical applications, the optimiztaion techniques have become a vital priority because of its wonderful properties. Optimization holds an important place in machine learning as it helps to improve the covergence rate, can enhance the degree of approximation, has the capability to select the features efficiently, etc. The fuzzy codes or values are optimized, and the best values are selected before classification. Four different types of optimization are utilized in this work such as Differential Search (DS), Shuffled Frog Leaping Algorithm (SFLA), Wolf Search (WS) and Animal Migration Optimization (AMO).

### 4.1. Differential Search Optimization

Then, the fuzzy modeled values are optimized again with the help of DS optimization developed by the Civicioglu [38]. In this algorithm, the search space is simulated as the food areas, and every point in the search space matches to an artificial superorganism migration. Finding the global optimal solution is the main goal of this migration. The algorithm begins with a randomly initiated artificial organism that utilizes the NP*D-dimension parameter vector with its respective minimum and maximum bound conditions expressed as follows:

(2) $${\vec{A}}_{\min}=\left\{a_{1,\min},a_{2,\min},\ldots ,a_{D,\min}\right\}$$

(3) $${\vec{A}}_{\max}=\left\{a_{1,\max},a_{2,\max},\ldots ,a_{D,\max}\right\}.$$

The $y^{th}$ component of the $z^{th}$ vector is generated as:

(4) $$a_{y,z,0}=a_{y,\min}+rand_{z,y}+[0,1].\left(a_{y,\max}-a_{y,\min}\right)$$

where $rand_{z,y}[0,1]$ is a uniform distribution random number between 0 and 1.

Assume z=1,…,NP and y=1,…,D. Between the artificial organisms, stop over vectors $s_{z,H}$ are generated that can be explained by a Brownian-like random walk model. Corresponding to each and every population individual, the algorithm creates a stopover vector in the current population. The technique for providing a stop over vector is expressed as follows:

(5) $$s_{z,H}=A_{z,H}+scale.\left(A_{r_{1},H}-A_{z,H}\right)$$

where $r_{1}\in \left[1,\ldots ,NP\right]$ are the chosen integers in a random fashion and $r_{1}\neq z$. The individual positions of the artificial organisms are controlled by the scale here, and the value of it is generated by a gamma random number, which in turn is controlled by a uniform distribution number ranging between 0 and 1. The individuals of the artificial organism of the super organism are used to calculate the search process of the stop over site and are expressed as

(6) $$s_{z,y,H}^{\prime }=\{\begin{array}{c} s_{z,y,H\begin{array}{cc} & if\begin{array}{cc} & r_{z,y}=0 \end{array} \end{array}}\\ A_{z,y,H\begin{array}{cc} & if\begin{array}{cc} & r_{z,y}=1 \end{array} \end{array}} \end{array}$$

where y=[1,…,D]; $r_{z,y}$ is an integer number either belonging to 0 or 1. $s_{z,y,H}^{\prime }$ indicates the trial vector of the $y^{th}$ particle in the $z^{th}$ dimension at the $H^{th}$ iteration. To choose the next population, a selection operator is used between the artificial organism population and the stop over site population, (H=H+1). The selection operator is expressed as:

(7) $$A_{z,H+1}=s_{z,H},\begin{array}{cc} if & f\left(s_{z,H}^{\prime }\right)\leq f\left(A_{z,H}\right) \end{array}$$

(8) $$A_{z,H+1}=A_{z,H},\begin{array}{cc} if & f\left(s_{z,H}^{\prime }\right)>f\left(A_{z,H}\right) \end{array}.$$

The standard pseudocode for the algorithm is given in Pseudocode 1.
**Pseudocode 1:**begin Generation counter initialization H=0 Population of NP*D individuals $A_{y}$ is randomly initialized. Parameter initialization p1,p2 Fitness evaluation for each individual in P  While stopping criteria is not satisfied do   scale = rand h(2*rand)*(rand−rand)       for z=1 to NP do           Randomly select b≠z           $s_{y}=a_{z}+scale\times \left(a_{b}-a_{z}\right)$       end       r=rand(NP,D);       If rand < rand, then           If rand < p1 then           for z=1 to NP do               r(z,:)=r(z,:)<rand           end       else       $for\begin{array}{cc} i=1 & to\begin{array}{cc} & NP\begin{array}{cc} & do \end{array} \end{array} \end{array}$           r(z,randz(D))=0       end   end else for z=1 to NP do       d=randz(D,1,[p2.rand])       for $y=1\begin{array}{cc} \begin{array}{cc} & to \end{array} & size\left(d,2\right)\begin{array}{cc} & do \end{array} \end{array}$           r(z,d(y))=0       end   end end r=r>0; s(r)=A(r);   for $z=1\begin{array}{cc} to & NP\begin{array}{cc} do & \end{array} \end{array}$           Offspring evaluation $s_{z}$       If $s_{z}$ is better than $A_{z}$, then       $A_{z}=s_{z}$       end if   end for   Memorize the best solution achieved so far  end while end

Thus, the global optimum values of the fuzzy modeled technique are obtained through the DS optimization algorithm.

### 4.2. Shuffled Frog Leaping Algorithm (SFLA)

One of the famous swarm evolutionary algorithms is SFLA, which follows the pattern of frogs exchanging vital and useful information as separated or split memeplexes when searching for food [39]. The optimum solution is obtained by the combination of local search and global search in memeplex. Here, many frogs together compose a virtual memeplex where each frog represents a candidate solution. Many memeplexes are obtained after division from the population, where each memeplex contains a certain number of frogs. Various memeplexes have their own behavior and culture, which can be easily affected by each other. Once the local search has been implemented for specific times, the mixing up of the memeplexes is done to get a new solution, and as a result, the information can be exchanged globally among all the memeplexes. Unless the desired convergence is obtained, the local and global search are alternated, thereby expressing it as maximum number of iterations or the achievement of a certain convergence accuracy.

For the unconstrained function optimization, the solution steps are as follows:

(1) Beginning stage/initialization: ‘c’ candidate solution is generated in its possible structural domain $\Omega \subset {ℜ}^{D}$ (for a D-dimensional problem). The expression of the ‘c’ candidate solution is done as the initial swarm $Swarm=\left(A_{1},A_{2},\ldots ,A_{c}\right)$, where $A_{i}=\left(a_{i1},a_{i2},\ldots ,a_{iD}\right)$; here, the candidate solution is described by the $i^{th}(1\leq i\leq c)$ solution.
(2) Classification of Memeplex: The population is partitioned into Zmemeplexes as follows. Based on the fitness value, the frogs are allocated to the groups. To the first memeplex, the first frog that has the highest value is moved to it, and to the second memeplex, the second frog that has the second highest value is moved to it. Similarly, to the last memeplex, the movement of the $z^{th}$highest frog is done. Unless the allocation of the last frog is done to the memeplex, these operations continue. Ultimately, every memeplex now contains frogs. Thus, c=y×z.
(3) Local search idea: In the memeplex, identify the best frog, and it is named as $A_{b}$. The worst frog is identified as $A_{w}$, and the global best is identified as $A_{g}$. In the following strategy, the renewal of $A_{w}$, the memeplex is done by means of searching and is represented as
  (9) $$R_{i}=\left\{\begin{array}{c} \min\left\{\mathrm{int}\left[r\left(A_{b}-A_{w}\right)\right],R_{\max}\right\},r\left(A_{b}-A_{w}\right)\geq 0\\ \max\left\{\mathrm{int}\left[r\left(A_{b}-A_{w}\right)\right],R_{\max}\right\},r\left(A_{b}-A_{w}\right)\leq 0 \end{array}\right\}$$
  (10) $$A_{new_{w}}=A_{w}+R_{i}$$
  where $R_{i}$ is the renewing value of the specific step size. int[a] is the roundness of a, r= random number ranging from (0,1). The maximum distance allowing the movement of frogs is $R_{\max}$.

After updating, if there exists $A_{new_{w}}\subset \Omega$, then $A_{new_{w}}$ is substituted by $A_{w}$. Otherwise, the $A_{b}$ is replaced in Equation (9) with $A_{g}$. Using Functions (9) and (10), the new $A_{new_{w}}^{\prime }$ is computed. If $A_{new_{w}}^{\prime }\subset \Omega$ and $F\left(A_{new_{w}}^{\prime }\right)<F\left(A_{w}\right)$ are present, then $A_{new_{w}}^{\prime }$ is replaced for $A_{w}$, or else to replace $A_{w}$, a new candidate solution is arbitrarily generated. Unless the designed search time is reached, the ending of the iteration is not done.

Global Information Exchange: Once the completion of the local search is done, then the mixing of all the memeplexes is done into a final swarm. For the algorithm to be terminated, it must be satisfied by one of the following three conditions:

(a) The objective function value should reach an optimum value.
(b) The predefined value is reached quickly based on the total number of iterations.
(c) No remarkable progress is returned in the main objective function during the iteration process.

Thus, the global optimum values of the fuzzy modeled technique are obtained through the SFLA optimization algorithm.

### 4.3. Wolf Search Optimization

The grey wolves always cohabit together, and hunting is usually done in groups [40]. The process of seeking and hunting is done as follows:

(a) Once a prey is found out, they plan to track, chase, and approach it in the most feasible manner.
(b) Once the prey identifies some danger, it starts running. Then, the grey wolves chase and encircle it.
(c) The prey gets harassed by the grey wolves unless it inhibits the movement.
(d) The attack starts and the prey gets killed.

Analyzing the searching and hunting process of grey wolves, this optimization algorithm was designed. In the mathematical modeling, alpha (α) represents the fittest solution, beta (β) represents the second best solution, and delta (δ) represents the third best solution. The remaining candidate solutions are assumed to be omegas (ω). When the optimization (searching) process along with hunting is carried out, all the omegas would be guided by the three grey wolves. The iteration begins when a prey is found out. Then, the omegas would lead the alpha, beta, and delta wolves to search and encircle the prey. To explain the encircling behavior, three coefficients $\vec{X}$, $\vec{Y}$, and $\vec{Z}$ are proposed.

(11) $$\begin{array}{l} \vec{Z_{\alpha }}=\left|\vec{Y_{1}.}\vec{P_{x}}-\vec{P}(t)\right|,\\ \vec{Z_{\beta }}=\left|\vec{Y_{2}.}\vec{P_{\beta }}-\vec{P}(t)\right|,\\ \vec{Z_{\delta }}=\left|\vec{Y_{3}.}\vec{P_{\delta }}-\vec{P}(t)\right| \end{array}$$

where the current iteration is described by t. The position vector of the grey wolf is represented by $\vec{P}$ and

(12) $$\vec{P_{1}}=\vec{P_{x}}-\vec{X_{1}}.\vec{Z_{\alpha }}$$

(13) $$\vec{P_{2}}=\vec{P_{\beta }}-\vec{X_{2}}.\vec{Z_{\beta }}$$

(14) $$\vec{P_{3}}=\vec{P_{\delta }}-\vec{X_{3}}.\vec{Z_{\delta }}$$

(15) $$\vec{P}(t)=\frac{\vec{P_{1}}+\vec{P_{2}}+\vec{P_{3}}}{3}.$$

The parameters $\vec{X}$ and $\vec{Y}$ are a combination of the control parameter ′x′ and the random numbers $\vec{r_{1}}$ and $\vec{r_{2}}$.

Therefore,

(16) $$\vec{X}=2\alpha \vec{r_{1}}-\alpha$$

(17) $$\vec{Y}=2\vec{r_{2}}.$$

The control parameter x chases $\vec{X}$, and ultimately, it causes the omega wolves to run away from the dominant wolves such as α,β,γ. The grey wolves run away from the dominant ones if $\left|\vec{X}\right|>1$, and this implies that the omega wolves run away from the prey, thereby exploring more space termed as global search in the optimization process. The dominant ones are approached if $\left|\vec{X}\right|<1$, which implies that the omega values follow the dominants approaching the prey and is termed as local search in optimization. As the iterations are being carried out, the control parameter x is expressed to be linearly declined for a value of 2 to zero and is represented as

(18) $$\alpha =2\left(1-\frac{iterations}{N}\right)$$

where the maximum iteration number is expressed as N, and it is started at the beginning. The application of this algorithm to any given problem is expressed as follows.

(1) The knowledge of some elemental parameters is known initially
(2) The random initialization of the grey wolf packing out of the space domain is done
(3) The other dominant grey wolves help lead the pack in order to search, find, and encircle the prey.

Once the grey wolves encircle the prey, it stops its movement, thereby ending the search, and therefore, the attack begins. The procedure of it is explained in Pseudocode 2.
**Pseudocode 2:**  Initialize the grey wolf population $P_{j}(j=1,2,3,\ldots ,n)$  Initialize x,X and Y  Calculate fitness of every search agent  $P_{x}=$ the best search agent  $P_{\beta }=$ the second-best search agent  $P_{\delta }=$ the third-best search agent  While (t< maximum number of iterations)    For each search agent    Update the position of the current search agent    End for    Update x,X and Y    Calculate the fitness of all search agents    Update $P_{x},$$P_{\beta },$$P_{\delta }$    t=t+1  End while  Return$\;P_{x}$


Thus, the global optimum values of the fuzzy modeled technique are obtained through the WS optimization algorithm.

### 4.4. Animal Migration Optimization

It is divided into the migration of the animal process and the updation of the animal process [41]. The movement of the animal groups from the present position to a new position is simulated by the algorithm in the migration process. The probability technique is used for the updation of animals, and it is simulated by the algorithm during the population updating process.

#### 4.4.1. Animal Migration Process

Three rules should be obeyed by the animal in the animal migration process.

(a) The collision is to be avoided with the neighbor
(b) The movement should be in the same direction as the neighbors
(c) The neighbors should remain close to each other.

Generally, a topological ring-like structure is used to define the concept of the local neighborhood of a particular individual. For every dimension of the individual, the neighborhood length is set to be ten in our simulation. Based on the set of indices or vectors, the neighborhood topology can be either static or dynamic. Assuming that the index of a particular animal is j, then its neighborhood comprises of animals having indices j−2,j−1,j,j+1,j+2, respectively. For instance, if the index of the animals is 1, then the neighborhood consists of an animal that has indices of NP−1, NP, 1, 2, 3, etc. Once the construction of the neighborhood topology is done, one neighbor is selected randomly, and the position of the individual based on this neighbor is updated as shown in the formulae as represented by

(19) $$Z_{j,H+1}=Z_{j,H}+\delta \cdot \left(Z_{neighbourhood,H}-Z_{j,H}\right)$$

where $Z_{neighbourhood,H}$ is the present position of the neighborhood. With the Gaussian distribution, a random number generation produces δ, $Z_{j,H}$ is the current position of the $j^{th}$ individual, and $Z_{j,H+1}$ is the new position of the $j^{th}$ individual.

#### 4.4.2. Population Updating Process

During this process, the animals leaving the group and some animals joining the new population is simulated by the Algorithm 1. With a probability $P_{a}$, some new animals will be needed to replace the individuals. The quality of the fitness is used to determine the probability of it. Fitness is sorted in descending order, and therefore, the individual with the worst fitness is 1, and the individual with the best fitness is 1/NP.
**Algorithm 1:** Population Updating ProcessFor j=1 to NP do For l=1 to D do If $rand>P_{a}$ then $Z_{j,H+1}=Z_{r1,H}+rand.\left(Z_{best,H}-Z_{j,H}\right)+rand.\left(Z_{r2,H}-Z_{j,H}\right)$
End if End for End for

The randomly chosen integers, $r_{1},r_{2}\in \left[1,\ldots ,NP\right]$, where $r_{1}\neq r_{2}\neq j$. After the production of the new solution $Z_{j,H+1}$, it is then compared with $Z_{j,H}$, and the individual with a better objective fitness is chosen as follows:

(20) $$Z_{j}=\left\{ \begin{array}{cc} Z_{j,H} & if\;f(Z_{j,H})>f(Z_{j,H+1})\\ Z_{j,H+1} & otherwise \end{array}\right..$$

Thus, the global optimum values of the fuzzy modeled technique are obtained through AMO algorithm.

## 5. Classification Techniques

The seven hundred and fifty code words that are attained per patient through fuzzy models are at different risk-level representations. Therefore, after passing through the above said optimization methods, the 750 code words are reduced to 375 code words by removing the redundant ones. The optimized values are finally fed to the classifiers for efficient classification. The classifiers used here are Logistic Regression (LR) [42], Fishers Linear Discriminant Analysis (FLDA) [43], K-Nearest Neighbor (KNN) [44], Support Vector Machine (SVM) [45], and Artificial Neural Network (ANN) based classifiers [46]. 

KNN: It is well-known that KNN is a very famous supervised learning technique which helps to trace testing sample’s class based on the K-nearest training samples majority class, and in our work, the value of K is reported to be 5.

ANN-Based Classifiers: For processing neurobiological signals extracted from EEG, ANNs are widely used. The ANN-based classifiers used in this work are Radial Basis Function (RBF) and Multilayer Perceptron (MLP).

RBF: It has 75 input neurons, 30 neurons in the hidden layer, and 1 output linear neuron.

MLP: There are 75 input linear neurons, one hidden layer with 25 neurons having hyperbolic tangent as the activation function, and 1 output neuron with logistic activation function.

SVM: It uses a non-linear kernel RBF with values ranging from 0.001 < γ < 0.01 and 1800 support vectors.

## 6. Results and Discussion

It is classified with a 10-fold cross-validation method, and the performance of it is shown in the tables below. The mathematical formulae for computing the Performance Index, Sensitivity, Specificity, and Accuracy is mentioned in the literature, and using the same, the values are computed and exhibited. Perfect Classification is represented as PC, Missed Classification is represented as MC, and False Alarm is represented as FA.

The Sensitivity is expressed as

(21) $$Sensitivity=\frac{PC}{PC+FA}\times 100.$$

Specificity is expressed as

(22) $$Specificity=\frac{PC}{PC+MC}\times 100.$$

Accuracy is expressed as

(23) $$Accuracy=\frac{Sensitivity+Specificity}{2}.$$

Performance Index (PI) is expressed as

(24) $$PI=\left(\frac{PC-MC-FA}{PC}\right)\times 100.$$

The Good Detection Rate (GDR) is expressed as

(25) $$GDR=\left(\frac{\left[PC-MC\right]}{\left[PC+FA\right]}\right)\times 100.$$

The Mean Square Error (MSE) is expressed as follows:

(26) $$MSE=\frac{1}{N}\sum_{i=1}^{N}{\left(O_{i}-T_{j}\right)}^{2}.$$

where $O_{i}$ indicates the observed value at a specific time, $T_{j}$ denotes the target value at model j; j = 1 to 28 for CVD patients and 14 for normal cases, and N is the total number of observations per patient in our case, which is 375. The training of the classifiers was implemented with a zero-training error of MSE.

Table 7 shows the average statistical parameters of various optimization techniques with different features for normal cases. As demonstrated in the Table 7, we can observe that there is no variation among the mean, variance, skewness, geometric mean, and harmonic mean parameters across the four optimization methods. Furthermore, it is inspired from the Pearson Correlation Coefficient (PCC) that the optimized values are non-linear and uncorrelated among the normal cases. The kurtosis parameter indicates diverged conditions for the four optimization methods. Sample entropy and approximate entropy attained higher values for the DS optimization method and low values at SLFA.

Table 8 shows the average statistical parameters at various optimization techniques with different features for CVD cases. As observed in the Table 8, we can identify that there is no variation among the mean, variance, skewness, geometric mean, and harmonic mean parameters across the four optimization methods. Furthermore, it is inspired from the PCC that the optimized values are non-linear and uncorrelated among the normal cases. The kurtosis parameter indicates variable conditions for the four optimization methods. Sample entropy attained higher values for the WS optimization method, and approximate entropy arrived at a high value in the SLFA. The AMO process settled at low values for sample entropy and approximate entropy.

Table 9 shows the Canonical Correlation Analysis (CCA) at various optimization techniques with different features for normal and CVD cases. It is widely acceptable that CCA values are more than 0.5, and it indicates a close correlation among the variables. As shown in Table 9, CCA values indicate that there is no correlation among the CVD and normal cases.

Table 10 indicates the consolidated results of accuracy (%) among the classifiers at various optimization techniques with different features for normal cases. In the DS optimization, MLP attained higher accuracy of 93.36%, and the Logistic Regression (LR) classifier arrived at a lower accuracy of 77.47%. As in the case of SLFA, the RBF classifier reached 92.45% accuracy, while the KNN classifier placed a low accuracy of 78.25%. For the WS optimization method, the RBF classifier maintained an accuracy of 94.79%, and here also, the KNN classifier is at a low accuracy of 78.33%. In the case of the AMO process, SVM–RBF attained a higher accuracy of 92.71%, and the LR classifier reduced to a low accuracy of 76.2%.

Table 11 shows the consolidated results of accuracy (%) among the classifiers at various optimization techniques with different features for CVD cases. In the DS optimization, SVM–RBF attained a higher accuracy of 93.75%, and the Logistic Regression (LR) classifier arrived at a lower accuracy of 81.51%. As in the case of SFLA, the RBF classifier reached 93.23% of accuracy, while the KNN classifier placed at a low accuracy of 82.29%. For the WS optimization method, the SVM–RBF classifier maintained an accuracy of 94.66%, and here also, the KNN classifier is at a low accuracy of 85.02%. In the case of the AMO process, SVM–RBF attained a higher accuracy of 95.05%, and the KNN classifier reduced to a low accuracy of 81.77%.

Table 12 shows the average performance index (%) among the classifiers at various optimization techniques with different features for normal cases. It is observed from Table 12 that for the DS optimization method, LR and FLDA have low PI values, and MLP has a high PI value of 84.66%. In the SLFA, KNN has the lowest PI of 23.01%, and the RBF classifier has a high PI of 82.18%. As in the WS optimization method once again, RBF attained a high PI of 88.38%, and the KNN classifier is at a low PI of 23.51%. For the AMO process, a high PI of 82.93% is arrived in the SVM–RBF classifier, and LR is ebbed at a low PI value of 9.12%. 

Table 13 shows the average Performance Index (%) among the classifiers at various optimization techniques with different features for CVD cases. It is observed from Table 13 that for the DS optimization method, LR has a PI value of 41.29%, and SVM–RBF has a high PI value of 85.7%. In SLFA, KNN has the lowest PI of 45.16%, and the RBF classifier has a high PI of 84.315%. As in the WS optimization method, once again, SVM–RBF attained a high PI of 88.045%, and the KNN classifier has a low PI of 57.22%. For the AMO process, a high PI of 89.18% is arrived in the SVM–RBF classifier, and the KNN classifier is ebbed at a low PI value of 42.58%. 

Table 14 denotes the average performance of parameters among the classifiers at various optimization techniques with different features for normal cases. As observed from Table 14, LR and KNN are the least preferred classifiers because of their low performance among all the parametric values such as accuracy, PI, GDR, and error rate. The RBF classifier is outperforming in the parametric value amongst all other classifiers across the four optimization methods.

Table 15 denotes the average performance of parameters among the classifiers at various optimization techniques with different features for CVD cases. As observed from Table 15, the KNN classifier is the least preferred classifier because of its low performance among all the parametric values such as accuracy PI, GDR, and error rate. The SVM–RBF classifier outperforms in the parametric values of all other classifiers across the four optimization methods.

## 7. Conclusion and Future Work

In the microvascular tissue bed, to detect the blood volume changes, PPG is used for the analysis of various disorders in the human body. With the help of a pulse oximeter, a PPG is often combined so that the skin is illuminated, and the light absorption changes are measured. In this work, a new approach has been developed for the PPG signal classification, as no previous works in this fuzzy adopted methodology have been reported so far. The extracted parameters are initially modeled with the help of fuzzy techniques, and then four types of optimization are used to get the best optimized values. Finally, the optimized values are classified with suitable classifiers to get the best results. When fuzzy-inspired modeling is implemented with AMO optimization and classified with the Support Vector Machine–Radial Basis Function (SVM–RBF) classifier, a classification accuracy of 95.05% is obtained for CVD cases. The second-best classification results are obtained when fuzzy-inspired modeling is implemented with WS optimization and classified with RBF reporting an accuracy of 94.79%. The third-best classification results are obtained when fuzzy-inspired modeling is implemented with WS optimization and classified with SVM–RBF, reporting an accuracy of 94.66%. Future works aim to work with other classifiers, especially deep learning, for a better classification of CVD levels.

## Figures and Tables

**Table 1:** Representation of output risk level classifications.

Risk Level	Representation
Normal (N)	A
Low (L)	B
Medium (M)	C
High (H)	D
Very High (VH)	E

**Table 2:** Binary representation of risk levels.

Representation	Binary String	Weight	Probability
E	10000	16/31 = 0.516129	0.073732
D	01000	8/31 = 0.258065	0.036866
C	00100	4/31 = 0.129032	0.018433
B	00010	2/31 = 0.064516	0.009216
A	00001	1/31 = 0.032258	0.004608
	11111 = 31	Σ = 1	

**Table 3:** Parameter ranges for various risk levels for fuzzy linguistic sets.

Risk Levels	Very Low	Low	Medium	High	Very High
Parameters					
Energy	0–0.1	0.7–3.6	2.9–8.2	7.6–11	9.2–30
Variance	0–0.3	0.15–0.45	0.4–2.2	1.6–4.3	3.8–15
Approximate Entropy	0–1.8	1–2.2	2–3.6	3.2–5	4.3–12
Mean	0–2	1–5	4–10	7–16	15–28
Standard Deviation	0–2	1–4.5	4–9	7–11.6	10–13
Skewness	0–0.3	0.15–0.45	0.4–2.4	1.8–4.6	3.6–10
Kurtosis	0–0.05	0.025–0.1	0.09–0.4	0.28–0.64	0.54–1
Peak maximum	0–3	1–5.2	4–9.3	7–11.6	10–14.6

**Table 4:** Fuzzy Associative Matrix (FAM) table for energy vs. variance parameters.

**Energy**	**Fuzzy Sets**	**Variance**				
		**Very Low**	**Low**	**Medium**	**High**	**Very High**
	Very Low	N	L	x	x	x
	Low	L	L	M	x	x
	Medium	x	M	M	H	x
	High	x	x	H	H	VH
	Very High	x	x	x	VH	VH

**Table 5:** Fuzzy modeled output for two-minute duration of PPG signal in CVD case.

BEEEEED	ADECEEE	ACDBCEE	ADEEEEE	EDEDEEE
BCDAECE	BEEEEDD	DDECEEE	BCDBECE	CEEEEED
DEEBEEE	ADEBDDE	DDEEDDD	CEEBEEE	DDEDEDE
DDEEDDD	CEEAEEE	CDECEEE	EDDEDDD	BEEAEEE
CDECEEE	ECDEDDD	BEEAEEE	DDECEEE	ECDEDDD
AEEAEEE	DDECEEE	EDDEDDD	BEEAEEE	DDECEEE
BDEAEEE	BEEAEEE	DDECEEE	ECDEDDD	AEEAEEE
BDEAEEE	EBCECEC	BEECDDE	BDEBEBE	CDEDDDD
AEEAEDE	BEEBECE	CDEDDDD	AEEAEDE	BDEAECE
EEEEDEE	AEEAEEE	BEEAEEE	EEEEDEE	BEEAEEE
CDEAEDE	EEEEDEE	AEEAEEE	CDEAEDE	DEEEDED
AEEAEEE	BDEBEDE	DEEEEEE	AEEBEDE	BDEBEDE

**Table 6:** Rhythmicity and Hurst exponent analysis on fuzzy modeled output.

Parameters	CVD Cases	Normal Cases
Rhythmicity	0.102905	0.111619
Hurst Exponent	0.401	0.535

**Table 7:** Average parameters at various optimization techniques with different features for normal cases.

Features	Optimization Methods	Mean	Variance	Skewness	Kurtosis	Geometric Mean	Harmonic Mean	Pearson Correlation Coefficient	Sample Entropy	Approximate Entropy
Fuzzy-Inspired and Modeled Features	DS	0.338409	0.006599	−0.66892	0.333147	0.31886	0.302793	0.027919	6.0187	3.416
	SFLA	0.371224	0.003499	−0.51108	−0.22984	0.36823	0.360155	0.040848	5.4976	2.816
	WS	0.371667	0.003439	−0.56445	0.005272	0.36375	0.358953	0.011319	5.5058	3.103
	AMO	0.337304	0.006448	−0.90151	0.745083	0.3256	0.300528	0.042151	5.991	3.2457

DS means Differential Search; SFLA means Shuffled Frog Leaping Algorithm; WS means Wolf Search; AMO means Animal Migration Optimization; AMO means Animal Migration Optimization.

**Table 8:** Average parameters at various optimization techniques with different features for CVD cases.

Features	Optimization Methods	Mean	Variance	Skewness	Kurtosis	Geometric Mean	Harmonic Mean	Pearson Correlation Coefficient	Sample Entropy	Approximate Entropy
Fuzzy-Inspired and Modeled Features	DS	0.356827	0.004257	−0.53336	1.32084	0.348029	0.339244	0.033675	6.41	4.731
	SFLA	0.345056	0.005002	−0.24626	−0.43423	0.336894	0.326393	0.046436	6.367	4.962
	WS	0.354519	0.003382	−0.53748	1.421386	0.34366	0.339225	0.086153	6.471	4.631
	AMO	0.363913	0.003522	−0.80286	1.527042	0.14617	0.349313	0.040009	6.1032	4.073

**Table 9:** CCA at various optimization techniques with different features for normal and CVD cases.

Features	Optimization Methods	CCA
Fuzzy-Inspired and Modeled Features	DS	0.14305
	SFLA	0.1089
	WS	0.12193
	AMO	0.1191

**Table 10:** Consolidated results of accuracy (%) among the classifiers at various optimization techniques with different features for normal cases.

Features	Optimization Methods	LR	FLDA	KNN	RBF	MLP	SVM-RBF
Fuzzy-Inspired and Modeled Features	DS	77.47188	79.17	85.42	91.47406	93.36	93.23
	SFLA	84.08213	81.055	78.25563	92.45	89.85625	91.93
	WS	85.02813	88.025	78.33727	94.795	85.67875	91.67
	AMO	76.2025	84.08213	82.095	92.45	90.625	92.71

**Table 11:** Consolidated results of accuracy (%) among the classifiers at various optimization techniques with different features for CVD cases.

Features	Optimization Methods	LR	FLDA	KNN	RBF	MLP	SVM-RBF
Fuzzy-Inspired and Modeled Features	DS	81.51	85.84047	83.9845	92.19	90.625	93.75
	SFLA	87.10938	84.08213	82.29	93.23	92.19	90.49688
	WS	87.10938	92.19	85.02813	92.58	91.47406	94.66438
	AMO	88.8125	86.32813	81.77	92.97	86.32813	95.055

**Table 12:** Average Performance Index (%) among the classifiers at various optimization techniques with different features for normal cases.

Features	Optimization Methods	LR	FLDA	KNN	RBF	MLP	SVM-RBF
Fuzzy-Inspired and Modeled Features	DS	17.9325	28.59	58.83	79.43063	84.66125	84.315
	SFLA	53.27363	38.93031	23.01625	82.18125	78.4275	80.72125
	WS	57.22125	69.7275	23.51391	88.38	59.85	80.01
	AMO	9.128125	53.27363	44.1925	82.18125	76.92	82.93

**Table 13:** Average Performance Index (%) among the classifiers at various optimization techniques with different features for CVD cases.

Features	Optimization Methods	LR	FLDA	KNN	RBF	MLP	SVM-RBF
Fuzzy-Inspired and Modeled Features	DS	41.29	60.4875	52.8515	81.4325	76.92	85.7
	SFLA	65.2125	53.27363	45.16	84.315	81.4325	77.17125
	WS	65.2125	81.4325	57.22125	82.55563	79.43063	88.045
	AMO	74.32875	62.3175	42.58	83.6225	62.3175	89.18

**Table 14:** Average performance of parameters among the classifiers at various optimization techniques with different features for normal cases.

Features	Parameters (%)	LR	FLDA	KNN	RBF	MLP	SVM–RBF
Fuzzy-Inspired and Modeled Features	Performance Index	34.38888	47.63036	37.38816	83.04328	74.96469	81.99406
	Accuracy	80.69616	83.08303	81.02697266	92.79227	89.88	92.385
	GDR	61.39114	66.16368	62.05419	85.58453	79.75384	84.77
	Error Rate	38.60886	33.83632372	37.94581	14.41547	20.24616	15.23

GDR means Good Detection Rate.

**Table 15:** Average performance of parameters among the classifiers at various optimization techniques with different features for CVD cases.

Features	Parameters (%)	LR	FLDA	KNN	RBF	MLP	SVM-RBF
Fuzzy-Inspired and Modeled Features	Performance Index	61.51094	64.37778	49.45319	82.98141	75.02516	85.02406
	Accuracy	86.13531	87.11018	83.26815625	92.7425	90.1543	93.49156
	GDR	72.26633	70.92411	66.53739	85.485	80.30859	86.98219
	Error Rate	27.73367	25.78035344	33.46261	14.515	19.69141	13.01781

GDR means Good Detection Rate.
